# Dynamic Finite Element Analysis of Mobile Bearing Type Knee Prosthesis under Deep Flexional Motion

**DOI:** 10.1155/2014/586921

**Published:** 2014-07-17

**Authors:** Mohd Afzan Mohd Anuar, Mitsugu Todo, Ryuji Nagamine, Shunji Hirokawa

**Affiliations:** ^1^Interdisciplinary Graduate School of Engineering Sciences, Kyushu University, 6-1 Kasuga-koen, Kasuga 816-8580, Japan; ^2^Faculty of Mechanical Engineering, Universiti Teknologi MARA, 40450 Shah Alam, Selangor, Malaysia; ^3^Research Institute for Applied Mechanics, Kyushu University, 6-1 Kasuga-koen, Kasuga 816-8580, Japan; ^4^Sugioka Memorial Hospital, 3-6-1 Kashiiteriha, Higashi Ward, Fukuoka, Fukuoka Prefecture 813-0017, Japan; ^5^Biomechanics Research Center, Kyushu University, 744 Motooka, Nishi-ku, Fukuoka 819-0395, Japan

## Abstract

The primary objective of this study is to distinguish between mobile bearing and fixed bearing posterior stabilized knee prostheses in the mechanics performance using the finite element simulation. Quantifying the relative mechanics attributes and survivorship between the mobile bearing and the fixed bearing prosthesis remains in investigation among researchers. In the present study, 3-dimensional computational model of a clinically used mobile bearing PS type knee prosthesis was utilized to develop a finite element and dynamic simulation model. Combination of displacement and force driven knee motion was adapted to simulate a flexion motion from 0° to 135° with neutral, 10°, and 20° internal tibial rotation to represent deep knee bending. Introduction of the secondary moving articulation in the mobile bearing knee prosthesis has been found to maintain relatively low shear stress during deep knee motion with tibial rotation.

## 1. Introduction

Introduction of mobile insert is believed to decrease polyethylene (PE) wear and facilitate range of motion (ROM) as well as tibial axial rotation by appearance of second moving interfaces between tibial insert and tibial tray [1, 2]. The advantage of this feature, however, is still in doubt and remains in further investigation. To the best of our knowledge, there is no apparent evidence of superiority of one design over another revealed in previous short-term and midterm clinical studies [2–5]. In these studies, mobile and fixed bearing knee prostheses were analyzed based on various attributes including Knee Score, Function Score, maximum flexion, pain score, and ROM. This observation is supported by* in vitro* assessments through wear analysis which is unable to disclose any significant difference in wear rate between mobile and fixed bearing PE insert [6]. Though, this result is contradicting with work by McEwen et al. who adapted comparable method and found obviously lower wear rate in mobile insert in comparison to fixed bearing tibial insert [7]. Good agreement with this study, however, was addressed by Sharma et al. who compared* in vivo* contact stress in the mobile bearing with the fixed bearing prostheses. They concluded that mobile bearing design capable of maintaining high conformity results in lower contact stress in comparison to fixed bearing design [8].

This study attempts to compare kinetics behavior of tibial condylar between mobile bearing and fixed bearing PE insert under dynamic loaded deep knee bending and tibial rotation.

## 2. Method and Analysis

**Figure 1:**
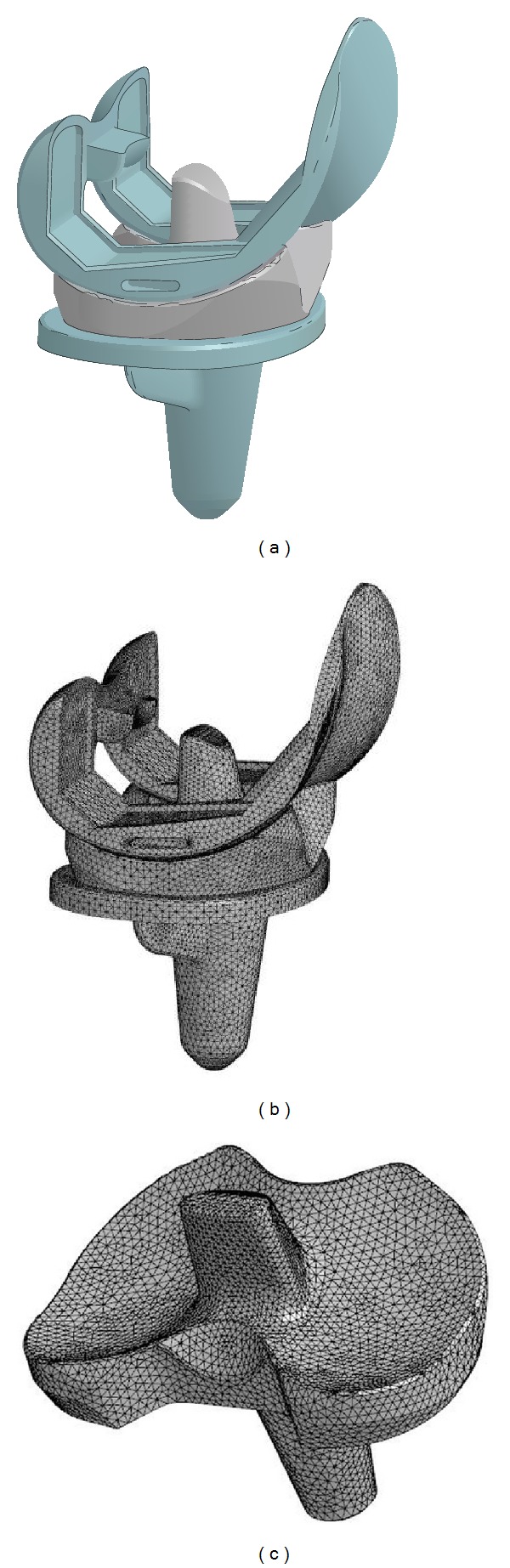
Computational models of mobile bearing type knee prosthesis. (a) CAD model, (b) Mesh model, and (c) Mesh model of tibial insert.

A 3D computational geometry of Japanese company commercially developed PS type mobile bearing knee prosthesis was used to construct a finite element (FE) model in FEMAP using 1.2 mm of edge length tetrahedron element (123,468 elements) with 32,436 nodes as shown in Figure 1. Femoral component and tibial tray were modelled as rigid bodies due to significantly higher Young's Modulus than mobile insert which was represented by an elastic-plastic material (*E* = 800 MPa) with Poisson's ratio of 0.40. The static and dynamic coefficients of friction of metal-on-plastic contact were selected as 0.04 [10, 11]. Penalty-based algorithm was used for contact definition. The FE model has been validated using NRG knee prosthesis model which applied similar model setup. The peak contact stress, mean contact stress, and contact area at 90° and 120° of flexion with neutral rotation of FE model were compared with results from previous work by Nakayama et al. [9].

The dynamic model was developed in LS-Dyna. Soft tissues constraint around the knee was represented by a pair of nonlinear springs inserted both anteriorly and posteriorly. The spring force in function of displacement can be expressed by

(1) $$\begin{array}{c} F=k_{1}d^{2}+k_{2}\;d=0.18667\;d^{2}+1.3313\;d, \end{array}$$

where *F* is the force exerted, *d* is the spring displacement, and *k*_1_ and *k*_2_ are the stiffness coefficients of the springs, respectively [12]. The combination of displacement and force driven knee joint was adapted to perform dynamic motion of the 6-degree-of-freedom prosthesis model. The femoral component was constrained in mediolateral (ML) displacement, anteroposterior (AP) displacement, and rotation, as well as proximodistal (PD) rotation, while being driven by flexional motion about ML-axis from 0° to 135° of angle. The vertical load from previous experimental work by Dahlkvist et al. was applied to the femoral component [13]. The tibial tray was constrained in PD displacement and ML rotation, while AP displacement was driven by AP force obtained from the same literature. At the same instance, the tray was set to perform axial rotation about PD-axis with neutral, 10°, and 20° of maximum tibial angle, respectively, to represent tibial rotation. Similar prosthesis model was used to represent fixed bearing design by fixing the mobile insert to the tibial tray to eliminate the effect of implant geometry.

## 3. Results

**Figure 2:**
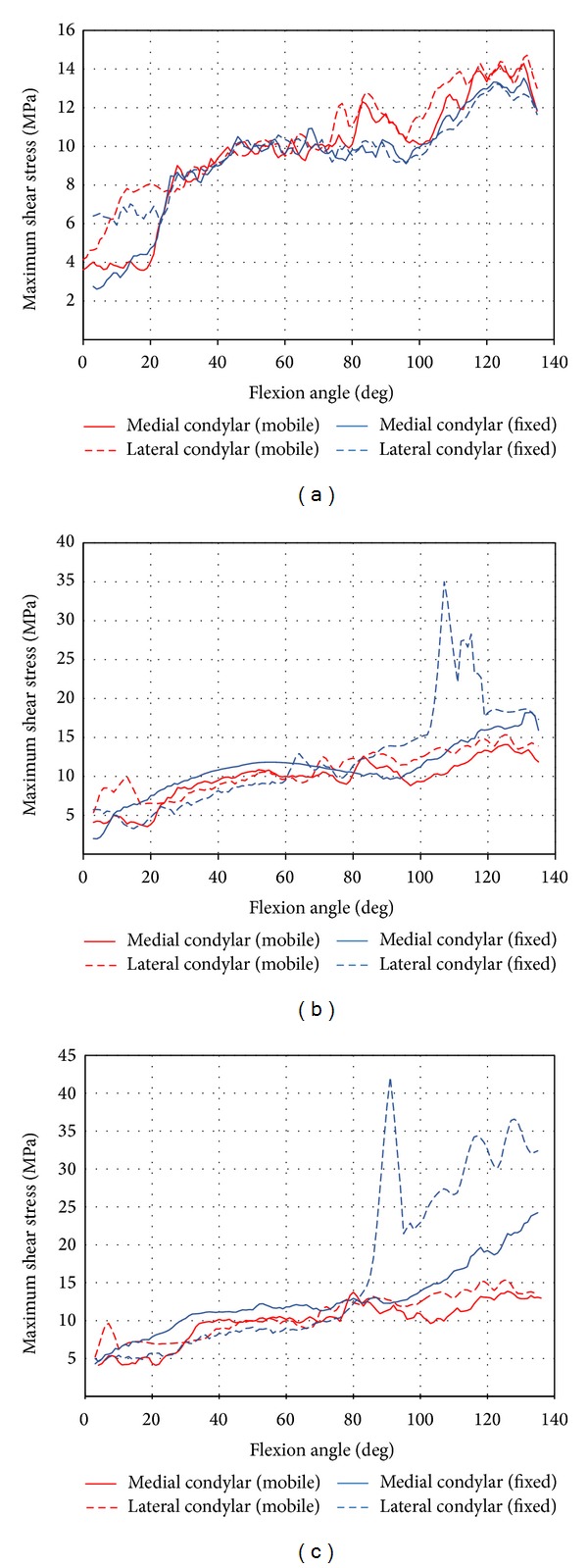
Maximum shear stress history from 0° to 135° of flexion angle. (a) Neutral position. (b) 10° tibial rotation. (c) 20° tibial rotation.

The comparison of results between FE model and experimental work by Nakayama et al. is shown in Table 1. The greatest differences of peak contact stress, mean contact stress, and contact area were 14.5%, 17.1%, and 7.9%, respectively, demonstrating a good agreement between both results. The maximum shear stress at medial and lateral tibia condyles in the function of flexion angle for neutral, 10°, and 20° tibial rotation, respectively, is illustrated in Figure 2. It was noted that the maximum shear stress at both medial and lateral condyles for neutral rotation increased with flexion angle. Similar trend was exhibited for the tibia which underwent 10° and 20° axial rotations. However, shear stress of mobile bearing insert was found less sensitive to tibial rotation in comparison to fixed bearing design where the maximum shear stress at both condyles varied from 5 MPa to 15 MPa for all conditions of tibial rotations.

**Figure 3:**
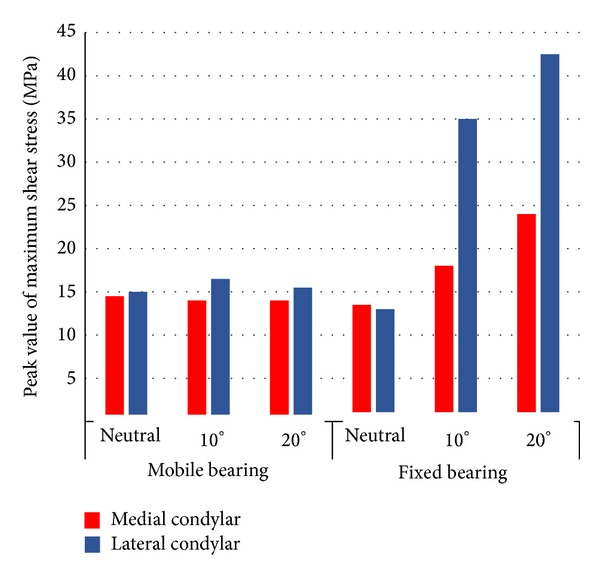
Peak values of maximum shear stress for mobile and fixed bearing TKA at neutral, 10°, and 20° internal tibial rotation.

Figure 3 shows the comparison of peak values of maximum shear stress between medial and lateral condyles with respect to tibial rotation and type of bearing mobility. Overall, peak values of maximum shear stress at lateral condylar were found relatively higher than medial condylar. As for fixed bearing insert which underwent axial rotation, the peak values at lateral condylar increased greater as compared with medial condylar. The peak values at medial condylar rose from 13.5 MPa with neutral position to 18 MPa and 24 MPa with 10° and 20° of tibial rotations, respectively. Meanwhile, at lateral condylar, increments from 13 MPa with neutral position to 35 MPa and 42.5 MPa with 10° and 20° of tibial rotations, respectively, were observed. On the contrary, for mobile bearing insert, the peak values remained around 14 MPa to 16 MPa at both condyles even subjected to axial rotational motion.

## 4. Discussion

### 4.1. Mechanics of Knee during Deep Flexional Motion

Most TKA postoperative patients especially in Asian countries may anticipate ability of deep kneeling which is associated with cultural and religious activities such as* seiza* among Japanese and kneeling in prayer for Muslims. Numerous studies have reported the critically large loadings generated at knee joint during deep bending motion. Dahlkvist et al. analyzed knee joint forces on six subjects and concluded that up to more than 5 times bodyweight of normal force is induced at tibiofemoral articulations during rapid descending [13]. In other investigations, single leg squatting was estimated to generate about 8% bodyweight of net normal force at knee joint [14]. Knee kinematics of high flexion analysis in previous works have showed the increasing tibial rotation up to 11° at 150° of flexion angle in intact knee and maximum of 17° at 137° of flexion angle in TKA postoperative knee [15, 16]. Excessive load combined with this state of motion may generate considerably high stress which, in turn, results in wear and delamination at articular surface of tibial insert as this study has shown relatively high shear stress, ranging from 10 to 50 MPa, induced at insert condylar during deep squatting.

### 4.2. Shear Stress States at Tibial Condylar

**Figure 4:**
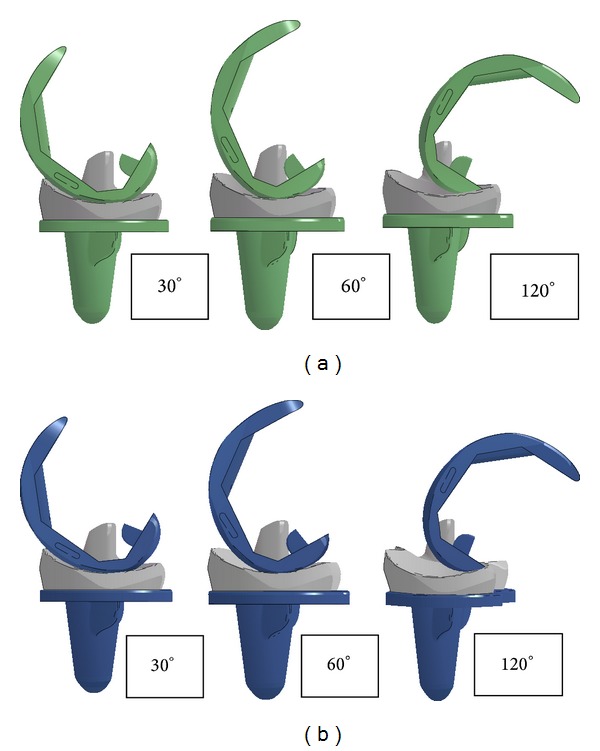
Lateral view of TKA during 30°, 60°, and 120° of flexion angle. (a) Mobile bearing. (b) Fixed bearing.

Wear and delamination of PE insert are among the most common problems in TKA that limit the survivorship of the prostheses. This defect is generated by excessive shear stress, contact pressure, and cross-shear associated with tibiofemoral contact geometry [15, 16]. Lower contact area in less conforming TKA results in higher contact pressure and shear stress relative to high conformity TKA. During femoral rollback, tibiofemoral contact shifts from larger contact area at centre of condylar to smaller contact area at posterior sides of both medial and lateral condyles leading to increasing shear stress with increasing flexion angle. Comparable mechanism happens in both mobile and fixed bearing TKAs as shown in Figure 4. As the knee flexes, medial femoral condylar moves anteriorly and lateral femoral condylar moves posteriorly causing internal rotation of tibia. Larger anterior surface relative to posterior on both tibia condyles leads to higher shear stress created at lateral condylar during deep bending motion. For fixed bearing condition, tremendous increment of shear stress at higher flexion angle (90°−110°) occurred due to impingement between femoral component and posterior articular surface of lateral condylar.

### 4.3. Mobile Bearing versus Fixed Bearing: Stress Sensitivity towards Knee Motion

**Figure 5:**
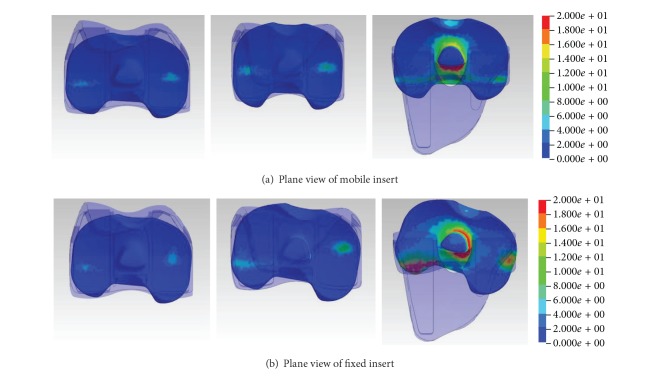
Shear stress distribution on the plane views of mobile and fixed inserts at 30°, 60°, and 120° of flexion angle, respectively, depicting the shear stress distribution. The fringe level is displayed in the unit of MPa.

Mobile bearing TKA is found less sensitive towards tibial rotation in terms of shear stress as compared with fixed bearing TKA. During tibia rotation, tibia transmits axial loading to tibial tray. In mobile bearing TKA design, kinematics of knee is uncoupled into two unidirectional motions by introducing second insert-tray articulations. Due to tibiofemoral engagement at proximal articular surface, the mobile insert maintains its neutral position and results in relatively lower shear stress at tibial condylar (Figure 5). In fixed bearing TKA, axial loading from tibial tray is transmitted evenly to tibial insert causing high shear stress generated on both medial and lateral condyles. Furthermore, multidirectional motion experienced by proximal surface of fixed insert will induce more wear and delamination. This result is supported by* in vitro* wear study by McEwen et al. who suggested that distribution of motions into femoral-insert and insert-tray interfaces in mobile bearing TKA has produced lower mobile insert wear defect [7].  Figure 5(b) illustrates the shear stress distribution in fixed insert at 30°, 60°, and 120°, respectively. It can be noted that the shear stress shifts from centre of condylar to posterior side of lateral condylar. However, research by Engh et al. revealed that appearance of second articulating surfaces introduced supplementary source of wear [17]. Existence of wear debris may alter the surface friction which is attributed to frictional force transmission from tibial tray to PE insert, hence affecting the stress states at upper side of tibial insert. This circumstance was not considered in the present study.

## 5. Conclusions

In conclusion, this study has showed the decomposition of multidirectional motion to unidirectional kinematics of femoral-insert and insert-tray articulating interfaces in mobile bearing TKA capable of maintaining lower stress at tibial condylar. Further investigation should be performed on various designs of mobile bearing TKAs to observe reproduction of this insert-tray mobility effect on stress states of tibial condylar.

## Figures and Tables

**Table 1:** Peak contact stress, mean contact stress, and contact area with neutral position at 90° and 120° of flexion angles.

Flexion angle (°)	Peak contact stress (MPa)		Mean contact stress (MPa)		Contact area (mm^2^)	
	FE model	Nakayama et al. [9]	FE model	Nakayama et al. [9]	FE model	Nakayama et al. [9]
90	27.3	25.9 ± 1.5	13.0	11.1 ± 0.2	42.6	45.1 ± 2.1
120	27.7	32.4 ± 0.5	12.4	14.8 ± 0.5	38.6	45.1 ± 2.1
